# Heterologous Expression of Xylanase Enzymes in Lipogenic Yeast *Yarrowia lipolytica*

**DOI:** 10.1371/journal.pone.0111443

**Published:** 2014-12-02

**Authors:** Wei Wang, Hui Wei, Markus Alahuhta, Xiaowen Chen, Deborah Hyman, David K. Johnson, Min Zhang, Michael E. Himmel

**Affiliations:** 1 Biosciences Center, National Renewable Energy Laboratory, Golden, Colorado, United States of America; 2 National Bioenergy Center, National Renewable Energy Laboratory, Golden, Colorado, United States of America; Universidad Autónoma del estado de Morelos, Mexico

## Abstract

To develop a direct microbial sugar conversion platform for the production of lipids, drop-in fuels and chemicals from cellulosic biomass substrate, we chose *Yarrowia lipolytica* as a viable demonstration strain. *Y. lipolytica* is known to accumulate lipids intracellularly and is capable of metabolizing sugars to produce lipids; however, it lacks the lignocellulose-degrading enzymes needed to break down biomass directly. While research is continuing on the development of a *Y. lipolytica* strain able to degrade cellulose, in this study, we present successful expression of several xylanases in *Y. lipolytica*. The XynII and XlnD expressing *Yarrowia* strains exhibited an ability to grow on xylan mineral plates. This was shown by Congo Red staining of halo zones on xylan mineral plates. Enzymatic activity tests further demonstrated active expression of XynII and XlnD in *Y. lipolytica*. Furthermore, synergistic action in converting xylan to xylose was observed when XlnD acted in concert with XynII. The successful expression of these xylanases in *Yarrowia* further advances us toward our goal to develop a direct microbial conversion process using this organism.

## Introduction

Yeasts are employed as the hosts of choices for the heterologous expression of proteins. In recent years, the “non-conventional” yeasts other than *Saccharomyces cerevisiae* have been receiving more attention in microbiological research. Among the “non-conventional” yeasts, *Y. lipolytica* is one of the most attractive and extensively studied model organisms for its genetic and physiological research [1], [2]. In addition, for its ability to secrete native and heterologous proteins at high levels [3]–[5], for example, wild type strains can secrete 1–2 g/l of alkaline extracellular protease (XPR2) [6], it has also been extensively used in a broad range of industrial applications. Furthermore, the availability of genome sequence of *Y. lipolytica* strain E150 (CLIB99) [7], [8] and the development of genetic tools such as transformation methods [9], and integrative expression cassettes [10]–[12] increase its suitability to be metabolically engineered.

*Y. lipolytica* has potential to become a unique model in developing biofuels. First, *Y. lipolytica* is known as oleaginous microorganism able to accumulate lipid intracellularly. Microbial lipid is considered to be an alternative feedstock to plant oil for biodiesel production [13]. We are interested in using this alternative lipid feedstock for direct conversion to renewable fuels. We are also interested in producing drop-in fuels and chemicals directly using oleaginous microorganisms. *Y. lipolytica* is reported to be able to metabolize glucose and xylose to produce lipid [14], [15]. However, most published oleaginous fungi cannot efficiently utilize lignocellulosic substrates due to lack of efficient cellulase and hemicellulase system. *Y. lipolytica* has a potent production and secretion machinery for both native and heterologous proteins [3]–[5], [16]–[18]. Heterologous expression of *Trichoderma reesei* endoglucanase II and cellobiohydrolase II has been reported successful in *Y. lipolytica* [19]. However, expression of xylanases in this yeast host has rarely been reported so far[20]. A xylanase TxXYN was reported displayed on the cell surface of *Y. lipolytica* but heterologous expression of xylanase genes inside *Y. lipolytica* especially secretion of xylanase proteins have never been reported. seWhile research is continuing on the development of a *Y. lipolytica* strain able to degrade cellulose, considering the capacity of protein production and secretion for *Y. lipolytica*, we are extending our initial studies of cellulase expression in *Yarrowia* to the expression of xylanases, as the ultimate utilization of lignocellulosic biomass relies on the combined action of cellulases and xylanases.

We are especially interested in two types of xylanases that are associated with degradation of xylan backbone [21], which are described as below: (1) endo-1,4-β-xylanase (EC 3.2.1.8), which randomly cleaves 1,4-β-D-xylosidic backbone linkages releasing xylo-oligosaccharides of variable lengths; (2) exo-1,4-β-xylosidase (EC 3.2.1.37), which successively removes D-xylose residues from the non-reducing ends of xylo-oligosaccharides and also cleaves xylobiose. Our criteria for selecting endo-xylanase and exo-xylosidase are that the enzymes should be originated from lignocellulolytic fungi with the optimal growth temperature at 30°C, the same growth temperature as that of *Yarrowia*. Preferably, the species and/or the targets of interest xylanases would be those that have been used for the commercial production of these enzymes. As a result, our selection of endo-1, 4-β-xylanases is the XynII from *Trichoderma harzianum*. In the case of exo-1,4-β-xylosidase, we chose XlnD from *Aspergillus niger*, which has been expressed in *S. cerevisiae* [22], [23] and *Aspergillus awamori* [24], and has been used by Sigma for commercial production of xylosidase.

In this study, we used Po1g, a derivative of the wild type *Y. lipolytica* W 29 and a high efficiency vector pYLSC for the heterologous expression of the above selected xylanase genes. A strong hybrid promoter hp4d and a secretion signal (*XPR2* pre region) were employed. The secreted target proteins were characterized and biochemical properties including the enzyme activities of the recombinant proteins were studied.

## Materials and Methods

### Microorganisms and vectors

*Yarrowia lipolytica* Po1g (MatA, leu2-270, ura3-302:URA3, xpr2-332, axp-2) and secretion vector pYLSC1 were purchased from Yeastern Biotech Co. (Taipei, Taiwan). *Yarrowia* Secretion Vector (pYLSC1, 7205 bp) contains the hybrid promoter (hp4d) and a secretion signal (XPR2 pre region: atgaagctcgctaccgcctttactattctcacggccgttctggcc, which encoded signal peptide MKLATAFTILTAVLA). It also contains a leucine selection marker gene (LEU2), which can complement the deletion of *LEU2* gene in the parent strain of Po1g.

### Constructs for expression of heterologous xylanase genes

Two constructs were built in the backbone of secretion vector pYLSC1, each carrying either of the following xylanase genes that encode the mature protein: XynII from *T. harzianum* and XlnD from *A. niger*. For each of the above genes encoding the mature protein, a *Sfi*I site (GGCCGTTCTGGCC) was added before the first codon of mature gene, and a *Kpn*I site (GGTACC) was added after the stop codon for the target mature proteins. The coding sequences of these genes were codon optimized based on the codon bias of *Y. lipolytica*, and were synthesized by DNA 2.0 (Menlo Park, CA).

The information describing the expressed genes and their corresponding *Yarrowia* transformants are summarized in Table 1.

**Table 1:** Xylanase genes expressed in *Y. lipolytica* using the secretion vector pYLSC1.

Proteins & source species	GenBank or Uniprot accession no.	amino acid no. of expressed enzymes (MW of secreted mature protein)	Transformant no., and recombinant yeast strain designation
XynII; *T. harzianum*	ACF40831	190 aa (21 kDa)	131; Yl[ThXynII]
XlnD; *A. niger*	O00089	778 aa (85 kDa)	133; Yl[AnXlnD]

### Transformation and selection

To prepare *Y. lipolytica* cells for transformation, strains were cultured 20 h in YPD pH 4 liquid broth at 28°C, 220 rpm in baffled shaker flasks. For the selection of auxotrophic recombinants (Leu^+^), transformants were grown on solid YNB medium (2% glucose w/v, 0.67% yeast nitrogen base w/o amino acids, 1.5% agar), incubated at 28°C for 2∼4 days.

The *YLEX* expression kit used in this transformation was purchased from Yeastern Biotech Co., Ltd. The transformation of *Y. lipolytica* with the plasmid constructs above was conducted using *YLOS* One step Transformation system included in the *YLEX* expression kit. The *Y. lipolytica* cells grown on YPD liquid broth were harvested after 20 h culturing. The harvested cells were washed twice with sterile dd-H_2_O to delete all YPD residues and collected by centrifugation at 3000 rpm for 5 min, followed by resuspending the cells (∼7×10^7^ cells per tube) in a tube containing 100 µL of freshly prepared *YLOS* cocktail including *YLOS* buffer, dithiothreitol (DTT) solution, carrier DNA and linearized plasmid DNA. The tube was incubated at 39°C for 1 h and then the entire cocktail was spread on dry YNB selection plates, incubated at 28°C for 2∼4 days until the colony of transformants developed.

### Plate assay for xylanase enzyme activity of transformants

Substrate-containing (birchwood xylan ×0502 from Sigma) mineral plates were prepared for the assay of endo-1,4-beta-xylanase and exo-1,4-beta-xylosidase activities. The mineral growth medium in the plates consisted of: 5 g/L xylan, 2 g/L of K_2_HPO_4_, 1.4 g/L (NH_4_)_2_SO_4_, 0.6 g/L MgSO_4_·7H_2_O, 0.4 g/L CaCL_2_·2H_2_O, 0.005 g/L FeSO_4_·7H_2_O, 0.002 g/L MnSO_4_·H_2_O, 0.002 g/L ZnSO_4_, 0.004 g/L CoCL_2_·6H_2_O, and 15 g/L agar. The plates were incubated at 28°C for 3∼5 days until colonies developed. After incubation, the plates were stained with Congo red (0.1%) for 20 min, and then washed with 1 M NaCl. Duplicate plates were made and stained for each strain and the staining results were consistent on duplicate plates.

### SDS-PAGE analysis

Invitrogen NuPAGE Novex Bis-Tris Mini Gel was used for running SDS-PAGE, for which SeeBlue Plus2 Prestained Protein Standard (LC5925; Invitrogen, NY) were used as the markers. 20 µL (Approximately 8∼10 µg) of protein preparation (50× concentrated extracellular crude enzyme solution) was loaded into each well. Before loading into gel, the protein were denatured by a reducing agent (which included DTT) and heated to 70°C for 10 min. The gel was run at 200 V constant for 40 min. After the electrophoresis, the gel was fixed with acetic acid/methanol solution, stained with Coomassie Blue overnight, and destained with deionized, distilled water for 7 h.

### Enzyme production and activity assay

The expression of the xylanase enzymes in *Y. lipolytica* Po1g was performed in 1-L baffled shaker flasks containing 300 ml YPD liquid broth (pH 4.0), incubating at 28°C, 200 rpm. Thirty ml of seed-culture (also YPD media) was inoculated into fermentation media after one-day culture. The crude enzyme preparation was collected at 4 d by centrifuging whole broth at 5000 rpm. This flask experiment was run in triplicates and the activity data was the mean of three triplicates.

The endo-1,4-beta-xylanase activity was determined by the method described by Bailey et al [25]. Briefly, 0.5 mL of 1.0% w/v birchwood xylan (in 50 mM pH 5.0 citrate buffer) was incubated with 0.1 mL of enzyme solution under 50°C for 5 min and then 1.5 mL DNS solution was added to the test tube and the mixture was boiled in water bath for 5 min. After cooling to room temperature, the absorbance at 540 nm was measured. The enzyme blank was prepared in the same way except that 0.5 ml of the citrate buffer was added to the substrate solution instead of the substrate solution. The xylanase activity was reported as katal/mL. 1 katal was the amount of enzyme needed to produce 1 mol of reducing sugar (D-xylose equivalent) from xylan per second.

The exo-1, 4-beta-xylosidase activity was evaluated using *p*-nitrophenol β-D-xylopyranoside (*p*NPX) [26]. The concentration of *p*NPX in reaction mixture is 3 mM. The substrate (in 50 mM citrate buffer) was incubated with the cell culture under 50°C for 30 min and then 3 mL of 10% Na_2_CO_3_ was added to stop the reaction. The release of *p-*nitrophenol (PNP) was monitored by measuring the absorbance at 408 nm. The activity was also reported as katal/mL. 1 katal was the amount of enzyme needed to produce 1 mol of *p-*nitrophenol from substrate per second.

### Assessment of exo-1, 4-beta-xylosidase in converting xylo-oligomers

The ability of the XlnD to convert xylo-oligomers was initially evaluated on low DP (up to DP = 6) preparations purchased from Megazyme (Wicklow, Ireland). 0.5 mL of mixed solutions of xylobiose, xylotriose, xylotetrose, xylopentose and xylohexaose (1.5 mg/mL each in 50 mM citrate buffer) were incubated with 0.5 mL of enzyme solution (14 nkat/mL) for 2 h at 50°C and then analyzed for oligomeric and monomeric xylose on a Dionex ICS-3000 ion chromatograph using a Carbopac PA-100 analytical column and pulsed amperometric detection (HPAEC-PAD) [27]. The column and detector were held at 30°C for the entire 60 minute run time. The mobile phases containing NaOH (150 mM) and NaOAc (250 mM) were operated in a gradient mode with a flow rate of 1 mL/min. The experiments were performed in duplicates.

### Digestion of Birchwood xylan

The synergistic digesting ability of XlnD to convert xylo-oligomers resulting from XynII was determined by DNS method using birchwood xylan. 0.5 mL of 1.0% w/v birchwood xylan (in 50 mM pH 5.0 citrate buffer) was incubated with 13.96 nkat (i.e. 0.01 mL, 1396 nkat/mL) of XynII crude enzyme preparation plus 7 nkat (i.e. 0.5 mL, 14 nkat/mL) of Yl[AnXlnD] 96 h cell culture under 50°C for 5 min and then 1.5 mL DNS solution was added to the test tube and the mixture was boiled in water bath for 5 min. After cooling to room temperature, the absorbance at 540 nm was measured. The xylan digestion test by XynII enzyme or XlnD cell culture individually was prepared in the same way except that the citrate buffer was added to the substrate solution instead of XlnD cell culture or XynII enzyme solution. The experiments were run in triplicates. The data presented were the mean of the triplicates, and error bars represent standard deviation.

### Evaluation of growth of recombinant strains on birchwood xylan

Growth of recombinant strains was conducted by inoculating respectively 1 mL of 24 h seed culture into baffled shaker flasks containing 10 mL medium consisting of (g/L): AZCL-dyed birchwood xylan (Megazyme, Ireland), 10; (NH_4_)_2_SO_4_, 1.4; KH_2_PO_4_, 2.0; MgSO_4_ · 7H_2_O, 0.6; CaCL_2_ · 2H_2_O, 0.4; FeSO_4_ · 7H_2_O, 0.005; ZnSO_4_ · H_2_O, 0.0014; CoCL_2_ · 6H_2_O, 0.0036; MnSO_4_ · H_2_O, 0.0001; yeast extract, 1.0; peptone 1.5. The culture was incubated at 28°C, 200 rpm for 2 days. The experiments were run in duplicates.

### Digestion of xylan in pretreated corn stover

Corn stover used for this study was pretreated by alkaline peroxide. The composition of the pretreated stover was determined according to the NREL Laboratory Analytical Procedure [28]. Digestions were run on the pretreated corn stover (0.03 g) with 0.5 mL (1396 nkat/mL) of XynII crude enzyme preparation plus 4.5 mL (14 nkat/mL) of sonicated Yl[AnXlnD] culture cells. Digestions were also run with each individual enzyme at the same loading above. All digestions were run for 24 h at 50°C in 50 mM citrate buffer at pH 4.8; after which, the hydrolysates were sampled for analysis by HPLC. The xylo-oligomers were analyzed on Dionex ICS-3000 ion chromatograph as described above. The experiments were conducted in triplicates. The data shown were the mean of the triplicates and error bars represent standard deviation.

## Results and Discussion

### Xylanase transformants grown on xylan containing plates

The constructs built in the backbone of secretion vector pYLSC1, each carrying either of the XynII from *T. harzianum* or XlnD from *A. niger*, were successfully transformed into *Y. lipolytica* Po1g respectively with high transformation efficiency ∼3000 colonies/µg DNA.

**Figure 1:**
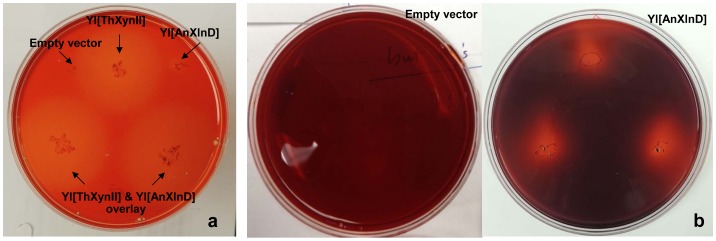
Growth of transformant Yl[ThXynII] and Yl[AnXlnD] on xylan mineral plate the plates were stained with 0.2% Congo red and destained by 1M NaCL.

Since endo-xylanase randomly cleaves 1,4-β-D-xylosidic linkages in xylan generating new reducing ends as well as some xylose (which *Yarrowia* cells can directly use) and exo-1,4-beta-xylosidase releases xylose from xylo-oligomers and xylobiose, we first characterized these transformants by testing their ability to grow on mineral plate with xylan as sole carbon source. The XynII transformant 131 (i.e. Yl[ThXynII]) grown two days on xylan-mineral plate exhibited halo zone around the colony after Congo red staining (Fig. 1a) while the empty vector control didn't show any halo zone, indicating the degradation of the xylan substrate by Yl[ThXynII] which therefore reflected the activity of the endo-xylanase; Compared to XynII transformant, the clear zone around the colony of XlnD transformant 133 (i.e. Yl[AnXlnD]) was small and the edge was obscure. Extending the incubation time of XlnD transformant to 4 days and staining the plate, more clear halo zone was observed indicating the activity of XlnD (Fig. 1b) but halo zone didn't appear for empty vector control even with the extension of incubation.

*Aspergillus* β-xylosidases are usually cell wall bound [29]. The different pattern of the clear zone as demonstrated by XlnD recombinant strain suggested the expressed enzyme was not well dispersed into the medium as the colony developed, which could be explained by the localization of the enzyme on the cell wall; In addition, the capacity of xylosidase to degrade xylan to reducing sugar, i.e. xylose, is limited, as verified by the xylan digestion test described below, which could also explain the small halo zone around the colony on the plate.

### SDS-PAGE analysis of xylanase proteins

**Figure 2:**
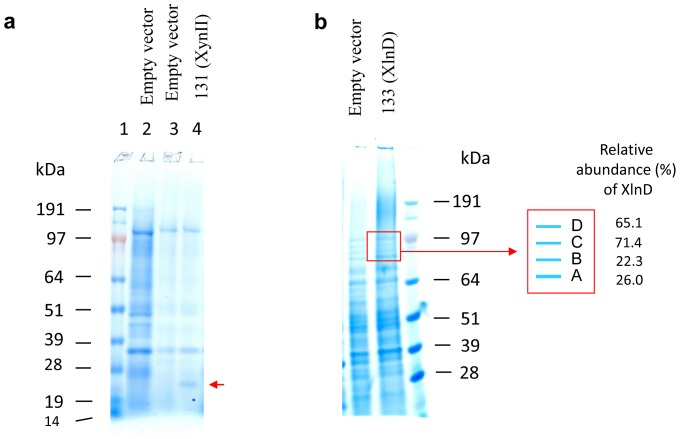
SDS-PAGE analysis of xylanases produced by *Y. lipolytica* transformants.

SDS-PAGE analyses of the enzymes produced by the recombinant strains are shown in Fig. 2. The identification of expressed target proteins on SDS-PAGE is based on the presence of new bands in each transformant, also based on the size of the expected target protein; as well as the comparison with the protein band pattern of empty vector control transformant. The expressed target protein bands on SDS-PAGE gel were further characterized by LC-MS, which proved the successful heterologous expression of XynII and XlnD in *Yarrowia*. Compared to the empty vector transformant, the sample prepared from the 85-fold concentrated enzyme solutions of XynII had extra band in the molecular weight size of approximately 22 kDa (Fig. 2a, which was almost the same as the theoretical size of the expressed protein (which is 21 kDa), suggesting this protein was likely less glycosylated.

Exo-β-1, 4–xylosidase (XlnD) is *N*-glycosylated and contains 15 potential N-glycosylation sites [30]. When XlnD was over-expressed in *A. niger and A. nidulans* [30], an hyper-glycosylated protein with molecular weight of 110 kDa was obtained. Based on the sequence study on glycosylation sites of XlnD, and also combined with our observation of no clear extra band on SDS-PAGE, we speculate XlnD was likely glycosylated to different levels in heterologous expression in *Y. lipolytica* (Fig. 2b). LC-MS analysis results (Table S1) revealed that all the four bands depicted as A, B, C and D were characterized to be XlnD with molecular weight of 91, 94, 96, 99 kDa, respectively. This agrees with our previous hypothesis on glycosylation and also helps explain the wide distribution of the expressed proteins on the gel instead of a single extra band compared to empty vector control. Considering the theoretical size of 85 kDa of XlnD, compared to XynII expression in *Yarrowia*, our results showed XlnD was glycosylated to a higher level due to more glycosylation sites in structure. The bands from the empty vector control as analyzed by LC-MS didn't hit with any target proteins although there were four bands appearing at similar positions on gel, indicating they were all endogenous proteins.

### Production of xylanase by transformants and their enzyme activities in the culture supernatant

For xylanase production and measurement of their enzyme activities, the XynII and XlnD transformants (transformant no. 131 and 133, respectively) were cultured in flasks and the enzyme activities of the culture supernatant/cells were measured as described in Materials and Methods. The culture supernatant of the XynII transformant showed strong xylanase activity as indicated by the release of reducing sugars. The cell culture of XlnD transformant also exhibited high activity against *p*NPX indicating the capacity of this enzyme to cleave xylobiose; however almost no activity was observed in culture supernatant indicating XlnD was not secreted extracellularly [29]. This result is in agreement with our observation of the weak halo zone of the XlnD transformant on plate assay, which can be explained by the localization of the XlnD protein on cell wall.

Relatively high levels of enzyme activities were recorded for these transformants at 72 h of growth. After 72 h, the enzyme activities increased gradually but not significantly. The endo-xylanase activity reached 1396 nkat/mL and exo-xylosidase reached 14 nkat/mL at 96 h respectively, which is comparable to the heterologous expression of endo-xylanase and exo-xylanase in *S. cerevisiae* which reported the endo-xylanase and exo-xylosidase activity obtained in shake flask were 1,577 and 5.3 nkat/mL respectively [22].

**Figure 3:**
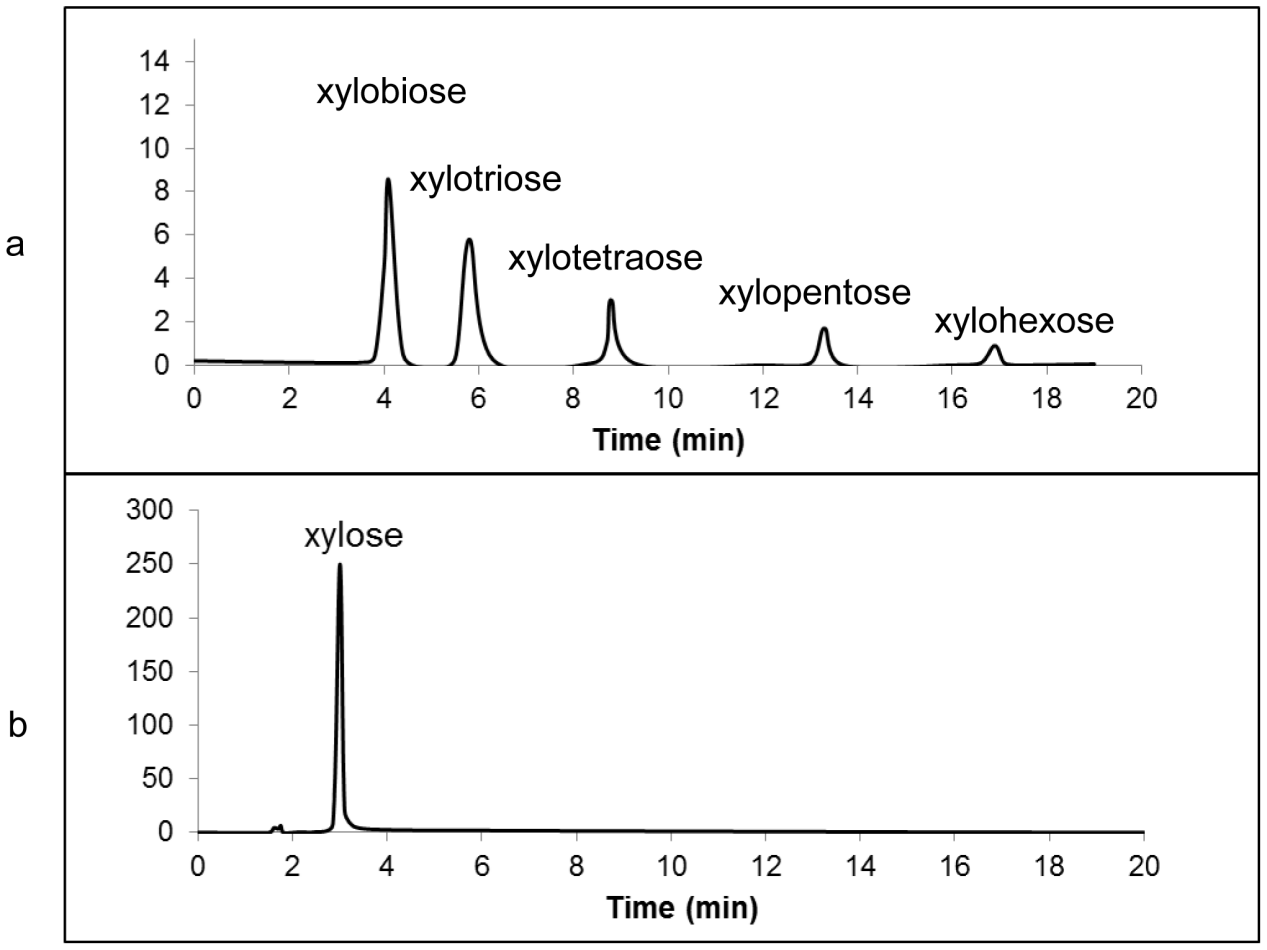
HPLC chromatogram of the digestion of mixed xylo-oligomers (degree of polymerization up to 6) by XlnD at 0 h (a), or 2 h (b).

The digestion of xylo-oligomers (with a degree of polymerization up to 6) with cell culture of XlnD demonstrated the enzyme ability to quickly hydrolyze small oligomers. XlnD was observed to completely hydrolyze 1.5 mg/mL solutions of individual xylo-oligomers to xylose within 2 h. Fig. 3 depicts the hydrolysis of a mixed solution of xylo-oligomers over a 2-h period. The efficacy of this enzyme made it possible to completely convert xylan to monomeric xylose by working on the xylo-oligomers generated by endo-xylanase. In addition, the ability of this enzyme to rapidly hydrolyze low molecular weight xylo-oligomers may also find its use in treating acid pretreatment hydrolysate which generally contains amounts of xylo-oligomers.

### Synergistic digestion of birchwood xylan with XynII and XlnD

**Figure 4:**
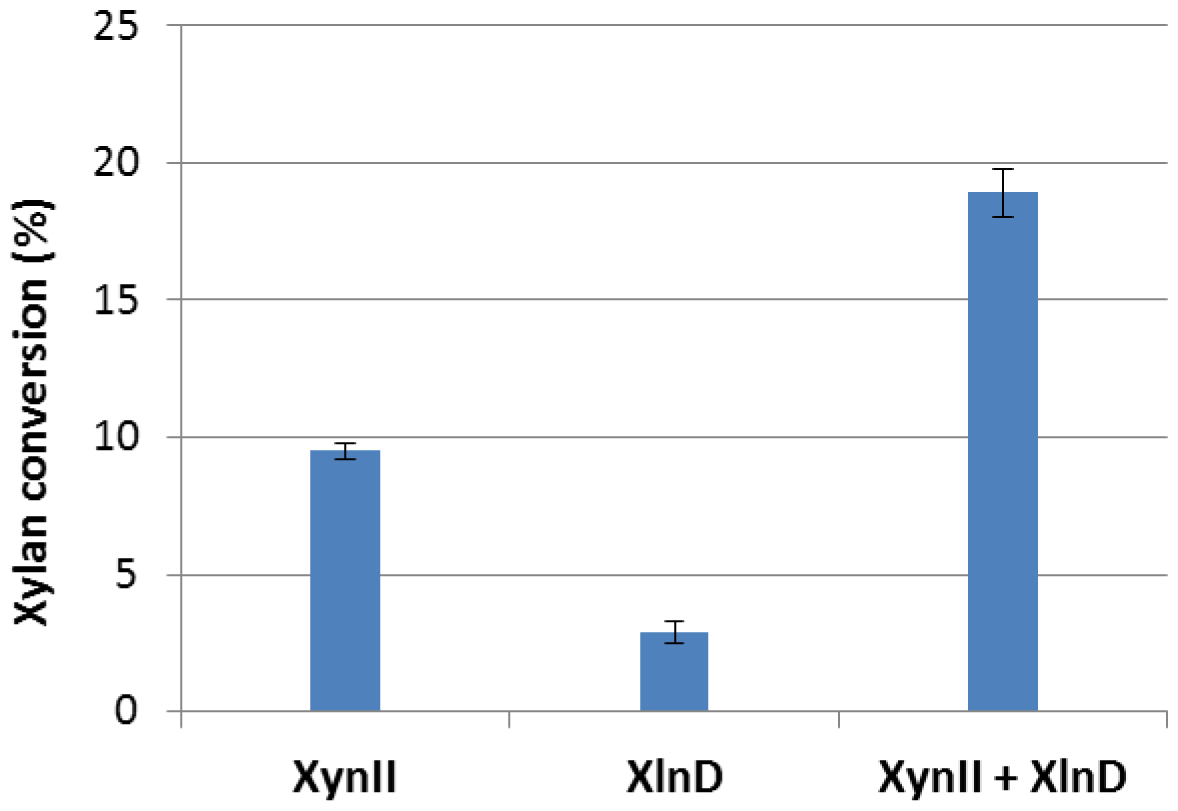
Xylan conversion by individual XynII, XlnD and combination of both enzymes after 5 min incubation. Error bars represent standard deviation of the triplicates.

**Figure 5:**
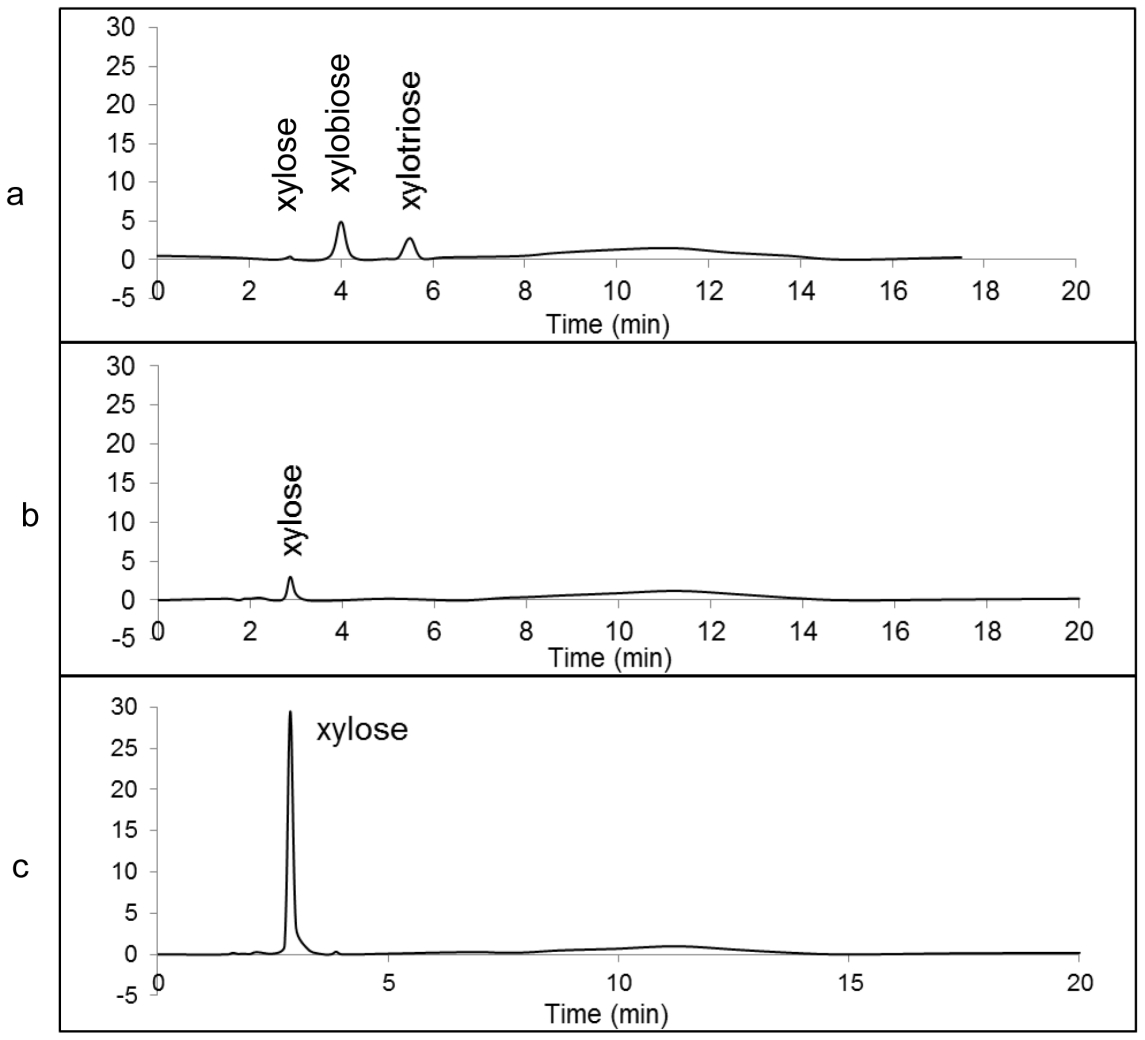
HPLC chromatograms of xylan digestion by individual XynII, XlnD and combination of both enzymes after 5 min incubation. (a) xylan digestion by XynII; (b) xylan digestion by XlnD; (c) Xylan digestion by combination of XynII and XlnD.

Digestions of birchwood xylan with XynII and the cell culture of XlnD showed the synergistic actions of the two enzymes on xylan (Fig. 4). After endo-xylanase enzyme (XynII) solution incubated with xylan solution for 5 min, about 10% of the xylan was digested whereas only 2.9% was digested by exo-xylosidase (XlnD). The digested products of xylan by endo-xylanase proved to be xylo-oligomers mainly xylotriose and xylobiose as shown in chromatograms of Figure 5(a) and the digested products by XlnD was small amount of xylose (Fig 5b). However, when these two enzyme worked together, an enhancement of xylan conversion of 18.9%, was achieved and the digested product was mainly the monomeric xylose (Fig. 5c) Furthermore, we observed the complete conversion of the xylan to monomeric xylose after 24 h incubation, which clearly showed the key role played by XlnD and that the final hydrolyzed product of xylan was xylose. The ability of XlnD to digest xylo-oligomers generated by XynII enhanced the total xylan conversion.

### Growth of xylanase transformants on birchwood xylan

**Figure 6:**
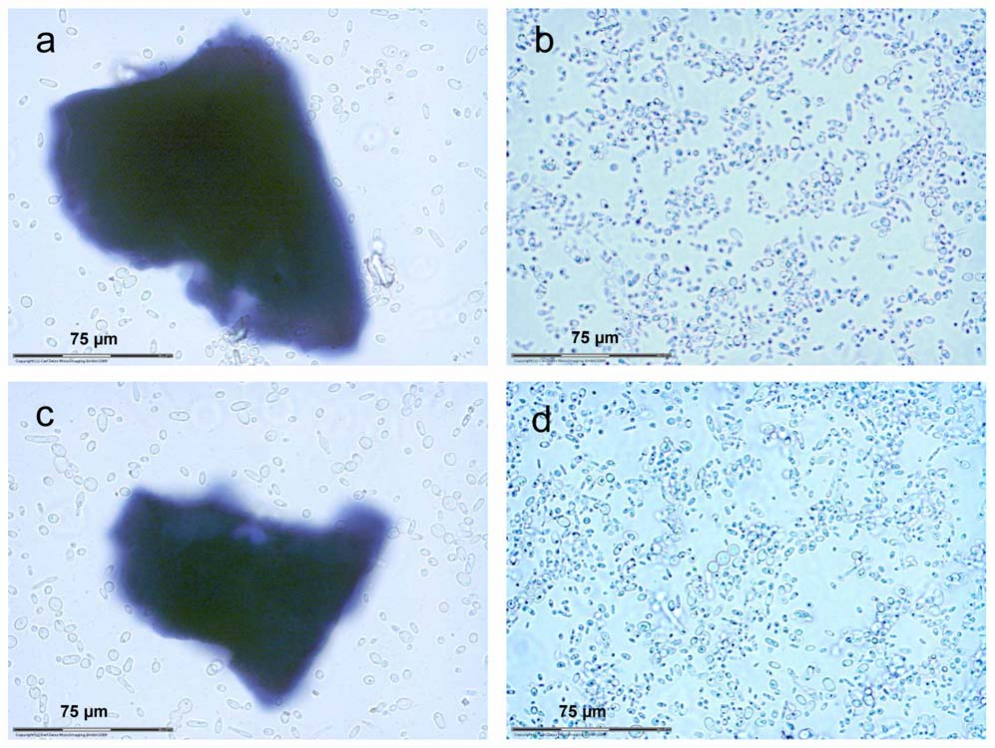
Growth of xylanase transformants in liquid medium with birchwood xylan as the carbon source. (a) Empty vector, (b) Yl[ThXynII], (c) Yl[AnXlnD], and (d) Yl[ThXynII] and Yl[AnXlnD].

In order to evaluate the degradation of xylan by xylanase transformants, the transformants were cultured in liquid media with dyed birchwood xylan as sole carbon source. Microscopic observation of the yeast growth and xylan decomposition was carried out using laser dissection microscopy (Carl Zeiss AG, Germany). The difference in the growth of cells among these yeast recombinant strains was demonstrated by cell number and size of xylan particles as shown in Fig. 6. We used Cellometer Vision (Nexcelom, Lawrence, MA) to quantitate the cell growth. Compared to XynII transformant (Fig. 6b, 6.60±0.02×10^8^ cells/mL) and combined cell culture (Fig. 6d, 1.05±0.01×10^9^ cells/mL), much less growth of cell was observed for the XlnD transformant (Fig. 6c, 5.80±0.02×10^6^ cells/mL); Although we found better growth of cells in the culture broth of XlnD transformant compared to the empty vector control (Fig. 6a, 3.50±0.02×10^6^ cells/mL), however this cell growth is not significant. This indicates that the capacity of converting xylan to xylose by exo-1,4-beta-xylosidase alone was very limited, and more xylan was degraded to support the cell growth when working synergistically with endo-1,4-beta-xylanase, as depicted in Fig 6d. This observation agrees well with the results of the synergistic test above.

Another prominent observation from these digestions is based on the difference of the xylan particle size. The advantage of choosing dyed AZCL xylan as substrate in this experiment is that we could easily observe the degradation of xylan during incubation as indicated by the blue dye released into the media. While more cell growth was observed in culture broth of XynII transformant and mixed XynII and XlnD transformants, the xylan particles also disappeared as clearly shown in Fig. 6b and 6d. In comparison, considerable undigested xylan residues were left in the culture broths of XlnD transformant and empty vector control (Fig. 6c and 6a). For empty vector sample (Fig. 6a), the size of residue xylan particle we observed was 120±10 µm (length) ×110±10 µm (width); for Yl[AnXlnD] (Fig. 6c) sample, the size of the residual xylan particle was 85±10 µm (length) ×85±10 µm (width). While for Yl[ThXynII] sample (Fig. 6b) and combined enzyme sample (Fig. 6d), the particles were much smaller, i.e. 5±2 µm×5±2 µm.

**Figure 7:**
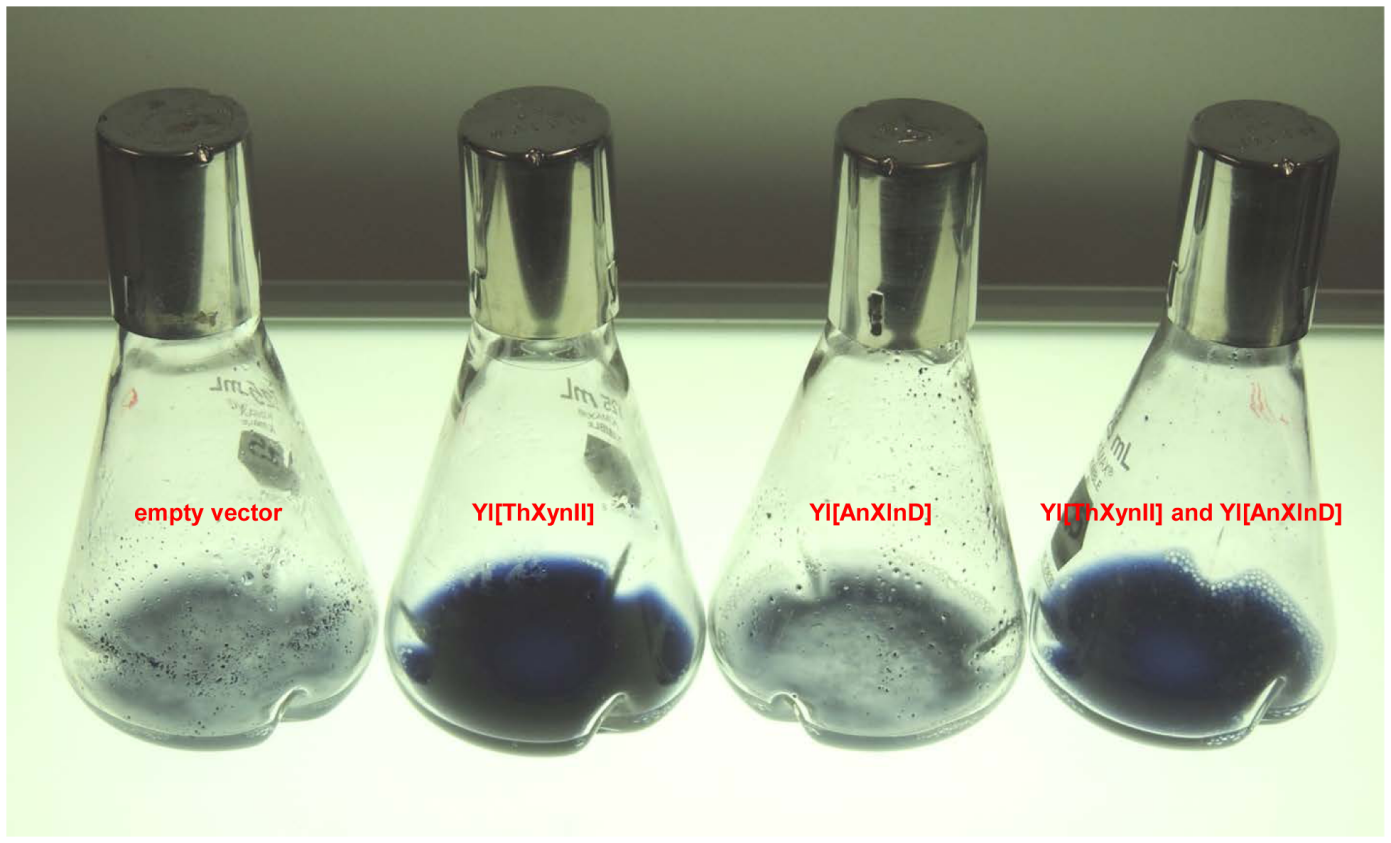
Growth of xylanase transformants in liquid medium with dyed AZCL-birchwood xylan as the carbon source. (a) Empty vector, (b) Yl[ThXynII], (c) Yl[AnXlnD], and (d) Yl[ThXynII] and Yl[AnXlnD].

This difference was even clearly seen just 30 min after the inoculating the seed culture, both culture broths of XynII only and mixed XynII and XlnD transformants became bright blue as blue dye released into the media as xylan degradation going on, whereas the blue xylan particles remained undigested in the broth of control and XlnD transformations (Fig 7).

### Digestion of xylan in pretreated corn stover

**Figure 8:**
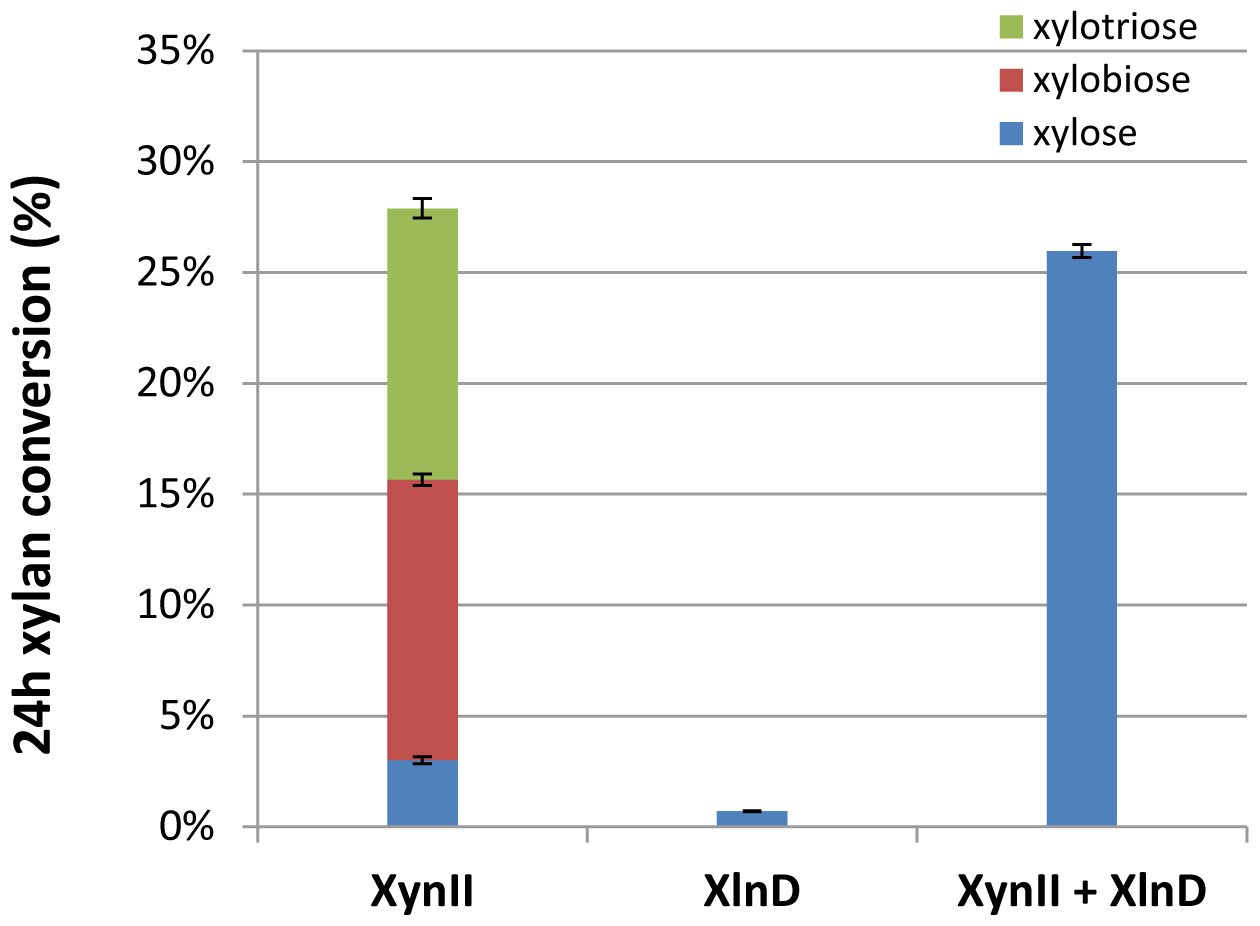
24h digestion of pretreated corn stover with individual and combination of both enzymes. Error bars represent standard deviation of the triplicates.

The previous digestion results on birchwood xylan showed XynII in presence of XlnD was able to digest birchwood xylan to monomeric xylose. For the pretreated corn stover substrate, as shown in Fig. 8, the XlnD contributed a lot to releasing monomeric xylose from xylan in biomass. This result was consistent with our observation of the two enzymes on birchwood xylan. XlnD had a significant effect on converting xylo-oligomers to monomeric xylose and the synergy of the two enzymes enhanced the complete conversion of xylan.

## Conclusions

In this study, we successfully demonstrated the heterologous expression of xylanases in *Y. lipolytica*. The expressed xylanase genes are XynII from *T. harzianium* and XlnD from *A. niger*. The obtained XynII and XlnD transformants showed the ability grow on xylan mineral plates, and also form halo zones on xylan mineral plates visualized by Congo Red staining. The SDS-PAGE analysis showed the xylanase proteins expressed in *Y. lipolytica* were likely sparsely glycosylated demonstrating *Y. lipolytica* is a good system for expression of heterologous proteins. The positive results from the enzymatic activity tests proved XynII and XlnD were successfully expressed in *Y. lipolytica* system and the expression levels were comparable to those expressed in *S. cerevisiae*. The expressed XynII was characterized by LC/MS to be the right protein. Synergistic action on converting xylan to xylose was observed when XlnD worked in concert with XynII. Our observation of the *Y. lipolytica* expression system is in accordance with the previous reports that XlnD was able to work on the xylo-oligomers generated by XynII, enhancing the xylan conversion to monomeric xylose. The successful expression of these xylanases in *Yarrowia* further advances us toward our goal to develop a direct microbial conversion process using this organism. Future work will direct to testing the synergistic action of the strains expressing xylanases developed together with strains expressing cellulases on pretreated biomass substrate. Ultimately, the combined expression of cellulases and xylanases in *Yarrowia* will result in a strain able to achieve direct microbial conversion of biomass to lipid, drop-in fuels and chemicals.

## Supporting Information

Table S1 **LC-MS characterization for expressed XlnD proteins from Yl[AnXlnD].**
(XLS)

## References

[1] Barth G, Gaillardin C, Wolf K (1996) Nonconventional yeasts in biotechnology, a handbook. Recherche 67:02.

[2] Barth G, Gaillardin C (1997) Physiology and genetics of the dimorphic fungus Yarrowia lipolytica. FEMS microbiology reviews 19:219–237. 9167256 10.1111/j.1574-6976.1997.tb00299.x

[3] Domínguez Á, Fermiñán E, Sánchez M, González FJ, Pérez-Campo FM, et al (2010) Non-conventional yeasts as hosts for heterologous protein production. International Microbiology 1:131–142. 10943351

[4] Madzak C, Gaillardin C, Beckerich JM (2004) Heterologous protein expression and secretion in the non-conventional yeast *Yarrowia lipolytica*: a review. Journal of Biotechnology 109:63–81. 15063615 10.1016/j.jbiotec.2003.10.027

[5] Müller S, Sandal T, Kamp-Hansen P, Dalbøge H (1998) Comparison of expression systems in the yeasts Saccharomyces cerevisiae, Hansenula polymorpha, Klyveromyces lactis, Schizosaccharomyces pombe and Yarrowia lipolytica. Cloning of two novel promoters from Yarrowia lipolytica. Yeast 14:1267–1283. 9802206 10.1002/(SICI)1097-0061(1998100)14:14<1267::AID-YEA327>3.0.CO;2-2

[6] Barth G, Gaillardin C, Wolf K (1996) Nonconventional yeasts in biotechnology. Yarrowia lipolytica Berlin: Springer.

[7] Dujon B, Sherman D, Fischer G, Durrens P, Casaregola S, et al (2004) Genome evolution in yeasts. Nature 430:35–44. 15229592 10.1038/nature02579

[8] Sherman D, Durrens P, Iragne F, Beyne E, Nikolski M, et al (2006) Genolevures complete genomes provide data and tools for comparative genomics of hemiascomycetous yeasts. Nucleic Acids Research 34:D432–D435. 16381905 10.1093/nar/gkj160 PMC1347522

[9] Chen DC, Yang BC, Kuo TT (1992) One-step transformation of yeast in stationary phase. Current Genetics 21:83–84. 1735128 10.1007/BF00318659

[10] Davidow LS, Apostolakos D, O'Donnell MM, Proctor AR, Ogrydziak DM, et al (1985) Integrative transformation of the yeast Yarrowia lipolytica. Current Genetics 10:39–48.

[11] Juretzek T, Le Dall MT, Mauersberger S, Gaillardin C, Barth G, et al (2000) Vectors for gene expression and amplification in the yeast Yarrowia lipolytica. Yeast 18:97–113. 10.1002/1097-0061(20010130)18:2<97::AID-YEA652>3.0.CO;2-U 11169753

[12] Dall MT, Nicaud JM, Gaillardin C (1994) Multiple-copy integration in the yeast Yarrowia lipolytica. Current Genetics 26:38–44. 7954894 10.1007/BF00326302

[13] Ratledge C, Wynn JP (2002) The biochemistry and molecular biology of lipid accumulation in oleaginous microorganisms. Advances in applied microbiology 51:1–51. 12236054 10.1016/s0065-2164(02)51000-5

[14] Papanikolaou S, Chatzifragkou A, Fakas S, Galiotou-Panayotou M, Komaitis M, et al (2009) Biosynthesis of lipids and organic acids by Yarrowia lipolytica strains cultivated on glucose. European Journal of Lipid Science and Technology 111:1221–1232.

[15] Tsigie YA, Wang CY, Truong CT, Ju YH (2011) Lipid production from Yarrowia lipolytica Po1g grown in sugarcane bagasse hydrolysate. Bioresource Technology 102:9216–9222. 21757339 10.1016/j.biortech.2011.06.047

[16] Cereghino GPL, Cregg JM (1999) Applications of yeast in biotechnology: protein production and genetic analysis. Current Opinion in Biotechnology 10:422–427. 10508632 10.1016/s0958-1669(99)00004-x

[17] Nicaud JM, Madzak C, Broek P, Gysler C, Duboc P, et al (2002) Protein expression and secretion in the yeast Yarrowia lipolytica. Fems Yeast Research 2:371–379. 12702287 10.1016/S1567-1356(02)00082-X

[18] Domínguez Á, Fermiñán E, Sánchez M, González FJ, Pérez-Campo FM, et al (2008) Non-conventional yeasts as hosts for heterologous protein production. International Microbiology 1:131–142. 10943351

[19] Boonvitthya N, Bozonnet S, Burapatana V, O'Donohue MJ, Chulalaksananukul W (2013) Comparison of the Heterologous Expression of Trichoderma reesei Endoglucanase II and Cellobiohydrolase II in the Yeasts Pichia pastoris and Yarrowia lipolytica. Molecular Biotechnology: 1–12. 10.1007/s12033-012-9557-0 22638966

[20] Duquesne S, Bozonnet S, Bordes F, Dumon C, Nicaud J-M, et al (2014) Construction of a Highly Active Xylanase Displaying Oleaginous Yeast: Comparison of Anchoring Systems. PloS one 9:e95128. 24743311 10.1371/journal.pone.0095128 PMC3990623

[21] Collins T, Gerday C, Feller G (2005) Xylanases, xylanase families and extremophilic xylanases. FEMS microbiology reviews 29:3–23. 15652973 10.1016/j.femsre.2004.06.005

[22] La Grange DC, Pretorius IS, Claeyssens M, van Zyl WH (2001) Degradation of xylan to D-xylose by recombinant Saccharomyces cerevisiae coexpressing the Aspergillus niger beta-xylosidase (xlnD) and the Trichoderma reesei xylanase II (xyn2) genes. Applied and Environmental Microbiology 67:5512–5519. 11722900 10.1128/AEM.67.12.5512-5519.2001 PMC93337

[23] Sun J, Wen F, Si T, Xu J-H, Zhao H (2012) Direct Conversion of Xylan to Ethanol by Recombinant Saccharomyces cerevisiae Strains Displaying an Engineered Minihemicellulosome. Applied and Environmental Microbiology 78:3837–3845. 22447594 10.1128/AEM.07679-11 PMC3346407

[24] Selig MJ, Knoshaug EP, Decker SR, Baker JO, Himmel ME, et al (2008) Heterologous expression of Aspergillus niger beta-D-Xylosidase (XlnD): Characterization on lignocellulosic substrates. Applied Biochemistry and Biotechnology 146:57–68. 18421587 10.1007/s12010-007-8069-z

[25] Bailey MJ, Biely P, Poutanen K (1992) Interlaboratory testing of methods for assay of xylanase activity. Journal of Biotechnology 23:257–270.

[26] La Grange DC, Pretorius IS, Van Zyl WH (1997) Cloning of the Bacillus pumilusβ-xylosidase gene (xynB) and its expression in Saccharomyces cerevisiae. Applied Microbiology and Biotechnology 47:262–266. 9114518 10.1007/s002530050924

[27] Qing Q, Wyman CE (2011) Hydrolysis of different chain length xylooliogmers by cellulase and hemicellulase. Bioresource Technology 102:1359–1366. 20943381 10.1016/j.biortech.2010.09.001

[28] Sluiter A, Hames B, Ruiz R, Scarlata C, Sluiter J, et al**.** (2008) Determination of structural carbohydrates and lignin in biomass. Laboratory Analytical Procedure.

[29] Pérez-González JA, van Peij NN, Bezoen A, Maccabe AP, Ramón D, et al (1998) Molecular cloning and transcriptional regulation of the Aspergillus nidulans xlnD gene encoding a β-xylosidase. Applied and Environmental Microbiology 64:1412–1419. 9546179 10.1128/aem.64.4.1412-1419.1998 PMC106163

[30] Peij NN, Brinkmann J, Vršanská M, Visser J, Graaff LH (1997) β-Xylosidase Activity, Encoded by xlnD, is Essential for Complete Hydrolysis of Xylan by Aspergillus Niger but not for Induction of the Xylanolytic Enzyme Spectrum. European Journal of Biochemistry 245:164–173. 9128738 10.1111/j.1432-1033.1997.00164.x

